# Clinical Papers on Ear Disease

**Published:** 1867-12

**Authors:** D. B. St. John Roosa

**Affiliations:** Prof. in the University Medical College


### CLINICAL PAPERS ON EAR DISEASE.

By D. B. St. JOHN ROOSA, M.D., Prof. in the University Medical College.

### NO. I.—INSPISSATED CERUMEN.

It is intended, in the papers which are proposed under the above title, to present some of the practical results of an experience in ear diseases, reaching over quite a large number of cases, in such a way that they may be useful as a guide to those who see comparatively little of the diseases of this organ.

Among the laity, and even in the profession, hardening of the ear-wax is regarded as quite a common and harmless affection. All forms of deafness are ascribed to this cause, and the first treatment that many ear patients receive, is a vigorous syringing to see "if the wax be not hardened," and this often without any preliminary examination. Impacted cerumen is indeed quite a common occurrence, but it is by no means as simple an affair as has been generally supposed. I do not mean by this, that it is anything more, as a general thing, than a local affection, but as such, it may produce results very detrimental to the function of hearing. It hardly seems to occur more frequently in persons with a soft skin than others, as has been suggested by some authors, for among the patients whom I have seen, careful examination has failed to detect any such origin. Persons with a dry and harsh skin have as often come to me with impacted cerumen, as the opposite class. A frequent cause is the too careful washing of the auditory canals with soap and water, which some over-clean persons delight in doing. This rinsing out the canal plugs the natural yellow wax, which is on its way out, down to the bottom of the canal, and being continued morning after morning, at last fills up the ear, and when the drum is once fairly covered, and pressed upon, and *not till then*, deafness results. It is somewhat remarkable how long persons may have the ears plugged up with hard wax without being aware of it. On examining persons who present themselves with impacted wax, only causing deafness on one side, we will nearly always find the same condition of things as to the wax, in the other ear. If the cerumen be very black and hard, and if it comes out in one large plug, we may conclude that it has been there for years. I recall two cases in which, from definite accounts, we could safely conclude that five years had elapsed since the deafness occurred. In both of these cases, the hearing became normal after the wax was removed. Impacted wax sometimes causes serious inflammation of the canal and drum. In one case, that of a young lady, suppuration of the drum resulted from hardened wax pressing upon it, and the wax was removed spontaneously like a shot from a pistol, and, as was stated, with almost as loud a report. This evacuation was preceded by the most intense pain. The removal of a plug three-fourths of an inch long from the other auditory canal, and which was wedged in very tightly, saved the patient from the inflammation which was so troublesome on the other side. In another case, still under treatment, what was supposed to be on first examination a plain case of inspissated cerumen, was found, after removal of the wax, to be one of inflammation of the integument which lines the canal. The removal of the hardened wax was, as it were, only the removal of a huge scab from an ulcerating surface. I have seen other cases like this.

Inspissated cerumen causes many symptoms. The prominent ones are:

1. Sudden deafness.
2. Tinnitus aurium.
3. Vertigo.
4. Earache.

Of course, an examination is the only method of clearing up the diagnosis. This examination should be undertaken with the ear mirror, (or otoscope, properly called,) and not with the syringe. In other words, it should be ocular, and not tactile. The trouble can hardly be confounded with any other affection. Wax which presses upon the drum is almost always black, not yellow, and nearly fills the canal. No decided prognosis can be given from seeing the wax, as to whether its removal will restore the hearing. Hardened cerumen very often forms over a perforated or ulcerated membrana tympani, and is then of course only a small part of the disease. It often results, also, from the dropping of oils into the ear for some therapeutical end seldom attained. The original disease for which the oils were used was then probably an affection of the cavity of the tympanum.

The habit of examining the ear in all cases with head symptoms, will sometimes assist materially in clearing up a diagnosis. I once cured a man from the effects of a supposed sun-stroke, by removing inspissated cerumen, who had been treated for two months in a hospital for cerebral disease.

Patients who have once had impacted wax, are apt to suffer again from the same cause, at least I have seen quite a large proportion of cases in persons who have been affected in the same way before. Such may be advised to have their ears syringed with a solution of bicarbonate of soda and water, about once in two months. The removal of the hardened mass is very often a tedious affair. I once spent an hour a day for a week in removing a mass from the ear of a lady patient. In the interim, the best solvents, such as soda, were used. With previous soaking the canal with a warm solution of soda, say a drachm to the half pint, ten minutes will generally suffice to remove the mass. A good india-rubber syringe, holding at least four ounces, should be used, and the auditory canal well straightened by holding up the auricle with the left hand, at the same time syringing with the right. The glass syringes are of no use. The stream sent in should be vigorous but steady, and care taken not to eject it with such force as to cause pain or dizziness. There should never be any pain caused in syringing the ear for any purpose. Where pain is produced, syringing will do harm. A thin bowl is held under the ear by the patient. No assistant is needed. No towel need be placed on the patient's neck, for, with careful manipulation, no water will be spilled.

The ear may contain an astonishingly great quantity of hardened ear wax, and an examination should be made very frequently during the course of the syringing to determine when it is all removed. No after-treatment is necessary. If, however, sounds are oppressive, as they often are, after the removal of large quantities of ear wax, a little cotton may be worn in the meatus for a day or two. The membrana sympani always appears reddened immediately after the removal of the cerumen, and then dull. It will be some days before it regains its normal translucency. If the hearing be not improved immediately on removing the wax, the middle ear should be inflated by Politzer's method. The drum is sometimes sunken in temporarily, and one or two passages of air through the eustachian tube will restore its position as well as the hearing.

Professor Gross recommends the use of a pick for the removal of impacted wax. This does very well as an aid where the wax is very hard. If it be used, the surgeon should have a mirror on his forehead, and never put the pick in the canal, unless he can see just what he is doing. Painful and even destructive inflammation may be caused by this mining out process. The general practitioner, to whom ear cases come in only a small proportion in his daily rounds, had much better rely on the use of a syringe and warm water where possible, having previously moistened the canal with a warmed solution of soda, zinc. sulph., or with glycerine and water, sweet oil, etc. Inspissated cerumen rarely occurs in children. I suppose there is no difference in the liability of the sexes, and I know of no well-established proximate cause, except the one given in the beginning of this article, *i.e.*, packing the meatus by the frequent pouring in of water. Yet, we might say that it is common for hardened wax to collect about a foreign body in the ear, such as a raisin, introduced originally to relieve earache, a cherry-pit, etc., but here the inspissated cerumen is only a concomitant. It is hardly to be credited, although formerly generally believed, that a diathesis has anything to do with it, or that there is any disease of the ceruminous glands. The cause is probably in one way or another mechanical—that is, there is some interference with the normal and daily removal of the secretion.—*Medical Record.*

